# Stillbirths by maternal-obstetric characteristics and Robson classification system: a cross-sectional study from eight district hospitals in Bangladesh

**DOI:** 10.7189/jogh.16.04077

**Published:** 2026-03-13

**Authors:** Lubna Hossain, Trisha Mallick, Abu Sayeed, Md. Lutful Kader, Md. Akib Al-Zubayer, Farhia Azrin, Hassan Rushekh Mahmood, Muna Shalima Jahan, Farhana Dewan, Md. Mahmudul Hassan Khan Shovon, Nondo Saha, Fariya Rahman, Md Refat Uz Zaman Sajib, Mohammad Delwer Hossain Hawlader, Dipak Mitra, Md Moazzem Hossain Sarker, Shams El Arifeen, Ahmed Ehsanur Rahman, Anisuddin Ahmed

**Affiliations:** 1 Maternal and Child Health Division, International Centre for Diarrhoeal Disease Research, Bangladesh, Dhaka, Bangladesh; 2 Khulna University, Khulna, Bangladesh; 3 Faculty of Health Sciences, McMaster University, Canada; 4 Dhaka Medical College and Hospital, Dhaka, Bangladesh; 5 Obstetrical and Gynaecological Society of Bangladesh, Dhaka, Bangladesh; 6 Department of Health and Kinesiology, University of Illinois Urbana-Champaign, Urbana, Illinois, USA; 7 Department of Public Health, North South University, Dhaka, Bangladesh; 8 Maternal, Newborn, Child & Adolescent Health, Directorate General of Health Services, Ministry of Health & Family Welfare of Bangladesh; 9 Department of Women's and Children's Health, Uppsala University, Uppsala, Sweden

## Abstract

**Background:**

Understanding maternal-obstetric determinants is key to stillbirth prevention. The Robson classification system enables monitoring and identification of high-risk groups. This study examined the prevalence and distribution of antepartum and intrapartum stillbirths by maternal-obstetric characteristics and Robson groups in district hospitals of Bangladesh.

**Methods:**

We conducted a cross-sectional analysis using prospectively collected data from Phases 4 and 5 (August 2022–June 2023) of a multi-phase implementation research study on the Robson classification system in eight district hospitals. All deliveries at ≥ 28 weeks of gestation were included, classified as live births, antepartum stillbirths (death before onset of labour), and intrapartum stillbirths (death after onset of labour). Descriptive analyses estimated stillbirth prevalence by timing, maternal and obstetric factors, and Robson groups. Associations were assessed using χ^2^ tests.

**Results:**

Out of 15 529 deliveries, 3.9% were stillbirths (56.6% intrapartum and 43.4% antepartum). Stillbirth prevalence varied across facilities (0.9–6.6%). A higher stillbirth burden was significantly associated (*P* < 0.05) with advanced maternal age (>34 years), preterm birth, lack of antenatal care, non-use of the partograph, and low birthweight. Breech presentation, induced labour, and vaginal delivery had the highest stillbirth percentage. Timing of stillbirth differed significantly by foetal presentation, onset of labour, and mode of delivery (*P* < 0.05). Stillbirth prevalence varied across Robson groups (0–17.5%). Intrapartum stillbirths predominated in Groups 1, 3, 6 and 8, while Group 10 was largely antepartum-dominated.

**Conclusions:**

Stillbirth remains a substantial burden in district-level hospitals in Bangladesh, with most deaths occurring during the intrapartum period and marked variation across facilities and Robson groups. The findings highlight preventable gaps in antenatal screening, labour monitoring, and timely emergency obstetric care. Application of the Robson classification system enables precise identification of high-risk obstetric groups for targeted quality-improvement interventions to reduce preventable stillbirths and accelerate progress toward national and global stillbirth reduction targets.

The World Health Organization (WHO) defines a stillbirth as the death of a baby occurring at or after 28 weeks of gestation but before or during delivery [1]. According to the United Nations Inter-agency Group for Child Mortality Estimation, approximately 1.9 million stillbirths occurred globally in 2021 – equivalent to 14 stillbirths per 1000 total births – with the vast majority occurring in low- and middle-income countries (LMICs), particularly in sub-Saharan Africa and South Asia [2]. Alarmingly, approximately three-quarters of these occurred in sub-Saharan Africa and Southern Asia, with LMICs bearing 84% of the global burden [2,3]. In South Asia, Bangladesh is among the countries with the highest stillbirth rates, reporting 21 stillbirths per 1000 live births in 2021 [4,5], far from the Every Newborn Action Plan (ENAP) target of 12 or fewer per 1000 live births by 2030 [6].

Stillbirths are commonly categorised as antepartum stillbirths (occurring before the onset of labour) and intrapartum stillbirths (occurring during labour and delivery) [7]. Antepartum stillbirths are most often associated with maternal complications such as infections, hypertensive disorders, anaemia, and poor antenatal care (ANC), whereas intrapartum stillbirths are largely linked to obstetric emergencies like obstructed labour, delayed caesarean section (CS), or foetal distress [7,8]. Evidence from South African Saving Babies 2008–2009 data showed that up to 75% of intrapartum stillbirths, and nearly 50% of antepartum stillbirths were associated with identifiable maternal conditions, which were the most common conditions among high morbidity mothers such as obstructed labour, hypertensive disease of pregnancy, preterm labour, antepartum haemorrhage, and maternal infections and chorioamnionitis [7,9,10]. Globally, intrapartum stillbirths represent more than 40% of all stillbirths, most of which are preventable through high-quality intrapartum monitoring and timely access to emergency obstetric care [3,6,11,12].

Accurate documentation and classification of stillbirths are essential for understanding underlying causes and targeting interventions. However, in many LMICs, including Bangladesh, weak health information systems and incomplete obstetric records hinder systematic analysis of stillbirth patterns [7,13–15], these typically analyse individual-level characteristics such as maternal age, parity, or gestational age, without adequately accounting for the clinical or health system context in which births occur. Consequently, the contribution of labour management practices and care quality to stillbirth remains poorly understood. The Lancet Series on Ending Preventable Stillbirths emphasised the need for a policy for improved data collection to guide policy and programme design [16].

The Robson ten-group classification system (TGCS) offers a practical and standardised framework for categorising all births into ten mutually exclusive groups based on key obstetric parameters such as parity, foetal presentation, gestational age, onset of labour, and mode of delivery [17]. While the TGCS is primarily used to assess CS trends, it can also be applied to stillbirth surveillance to identify high-risk groups and potential care-related determinants [18,19]. A recent study across 16 hospitals in sub-Saharan Africa involving more than 80 000 births demonstrated that using TGCS for stillbirths enables differentiation between biological risk and health system performance, revealing care patterns otherwise missed in conventional analyses [18]. Although Bangladesh has documented specific maternal-obstetric determinants of stillbirth, no study has systematically applied the Robson classification to identify which obstetric groups contribute most to stillbirths and where care gaps may exist [5,20,21].

Understanding these patterns can provide actionable insights to improve maternal and newborn outcomes by identifying high-risk groups and addressing facility-level gaps in care. Therefore, this study aimed to assess the prevalence and distribution of antepartum and intrapartum stillbirths using the Robson classification system and selected maternal-obstetric characteristics across eight district hospitals of Bangladesh.

## METHODS

### Study design and setting

We conducted a facility-based cross-sectional study using data from Phases 4 and 5 (August 2022 and June 2023) of the implementation research titled ‘Effect of an Integrated Intervention on the Availability and Completeness of Robson TGCS-Related Data in District Hospitals in Bangladesh’ [22]. The parent study, implemented from January 2021–June 2023 across eight district hospitals: Bagerhat, Bhola, Bogura, Gaibandha, Khagrachari, Munshiganj, Netrokona, and Sunamganj (Figure S1 in the **Online Supplementary Document**), followed five consecutive phases (Figure S2 in the **Online Supplementary Document**) [22]. Phases 1–5 focused on stakeholder consultation, introduction of Robson TGCS, development of the ‘Robson TGCS Report Form,’ capacity-building, a multi-level monitoring and supportive supervision system to improve data availability and completeness, while Phases 4–5 represented optimal data quality. These hospitals serve as secondary-level referral facilities with obstetric and anaesthesia teams. The facilities were purposively selected from each of the eight divisions of Bangladesh in consultation with the Maternal, Newborn, Child, and Adolescent Health Programme of the Directorate General of Health Services. Selection criteria included the availability of both vaginal and caesarean delivery services, and the presence of an Obstetrics and Gynaecology consultant paired with an anaesthesiologist.

### Robson classification system

We applied the Robson classification system to categorise all facility deliveries in the study hospitals which is a standardised, mutually exclusive, and totally inclusive system that classifies all women giving birth into 10 groups, with three broader subgroups, using six obstetric variables: parity (nulliparous or multiparous), history of previous CS (yes or no), foetal presentation or lie (cephalic, breech, or transverse), onset of labour (spontaneous, induced, or pre-labour CS), gestational age (preterm or term), and mode of delivery (vaginal delivery (VD) or CS) (Table S1 in the **Online Supplementary Document**) [23]. Deliveries with missing information on one or more required Robson variables were marked as ‘Unclassified’ and excluded from group-based analyses but retained in the total sample. These six obstetric variables are clinically relevant to stillbirths, as they collectively reflect maternal risk profiles, foetal conditions, and the quality of intrapartum care. Applying the Robson classification system, therefore, provides a structured and standardised approach to identify high-risk obstetric groups contributing to stillbirths within facility settings.

### Classification of stillbirths

Antepartum stillbirths were defined as foetal deaths occurring before the onset of labour, determined by the absence of foetal heart sounds (FHS) on admission, assessed by a skilled birth attendant using a handheld Doppler device. Intrapartum stillbirths were defined as foetal deaths occurring after the onset of labour, where FHS were present on admission but absent before birth. When FHS documentation was incomplete or uncertain, foetal appearance at birth was used as a supportive diagnostic criterion in accordance with the National Emergency Obstetric and Newborn Care (EmONC) register guidelines. As per instruction in the EmONC register guidelines, a fresh stillbirth was defined as foetal death occurring within 12 hours before delivery, where the newborn showed no signs of skin peeling or decomposition, indicating likely intrapartum stillbirths. A macerated stillbirth was defined as foetal death occurring more than 12 hours before delivery, with visible skin discolouration or tissue degeneration, indicating antepartum stillbirth. These classifications were based on information routinely recorded in the EmONC register and verified by the facility obstetrician to ensure consistency and minimise misclassification.

### Study population

This study population included all women who gave birth (live births and stillbirths) in the eight selected district hospitals (DHs) during the analysis period (August 2022, and June 2023). Inclusion criteria encompassed all deliveries of a baby born with no signs of life at ≥ 28 weeks of gestation. Exclusion criteria included deliveries with a gestational age below 28 weeks and those with missing information on the timing of stillbirth (antepartum or intrapartum). A total of 15 529 mothers with a gestational age of 28 weeks or more were included in the analysis (Figure S3 in the **Online Supplementary Document**).

### Data collection and validation

Data were extracted from the EmONC register and from the Robson TGCS Report Form maintained at each facility. Trained Field Research Officers visited the facilities monthly and extracted data after validation and approval by the consultant obstetrician, who verified the accuracy of the information recorded in the register books. Field Research Officers then photocopied the register books and sent them to the central office, where the validated data were entered into International Centre for Diarrhoeal Disease Research, Bangladesh (icddr,b’s) in-house Data Management System. Following data entry, the research physician and the statistician at icddr,b reviewed the data set for completeness, consistency, and accuracy before analysis.

### Exposure variables

The following demographic background and obstetrics characteristics of mothers who delivered live births and stillbirths at the selected DHs were used as exposure variables in this study. The variables were maternal age (< 20 years, 20–24 years, 25–29 years, 30–34 years, > 34 years), gestational age (preterm, term, post term), received ANC (Yes, No), partograph used (Yes, No), sex of the newborn (male, female), birthweight of the newborn (in kg) (< 2.5, 2.5–3.5, 3.6–4.5, and > 4.5), gravida (primigravida, multigravida, grand multigravida), foetal presentation (breech, cephalic), onset of labour (induced labour, pre-labour CS, spontaneous) and mode of delivery (normal VD, CS). These variables were chosen because they are routinely recorded in the national EmONC register and represent key demographic and clinical factors associated with stillbirths, as supported by both national and global evidence. The Robson classification system is further classified into 10 distinct and non-overlapping groups (Table S1 in the **Online Supplementary Document**) [23].

### Outcome variable

Our main outcome was to evaluate the prevalence and distribution of antepartum and intrapartum stillbirths using the Robson classification system and maternal-obstetric characteristics in selected district hospitals of Bangladesh. Stillbirths were categorised according to the WHO and Lancet Stillbirth Series definitions [1,7], with classification procedures and verification methods described in the Data Collection and Quality Assurance section.

### Data analysis plan

We examined a total of 15 529 deliveries across eight DHs. Each pregnancy outcome was categorised as live birth, stillbirths and further divided into antepartum and intrapartum stillbirths. We have performed descriptive cross-sectional analysis. Prevalence of antepartum and intrapartum stillbirths were calculated and characterised by maternal characteristics, obstetric factors and Robson classification groups. Additionally, bivariate analyses by χ^2^ tests were conducted to evaluate associations between stillbirth types and important maternal-obstetric variables and Robson groups. No multivariable or advanced statistical modelling was performed. The statistical analyses were performed with the use of the STATA 15 (StataCorp LLC, College Station, Texas, USA) and *R* statistical software version 4.4.2 (R Core Team, Vienna, Austria).

## RESULTS

**Figure 1:**
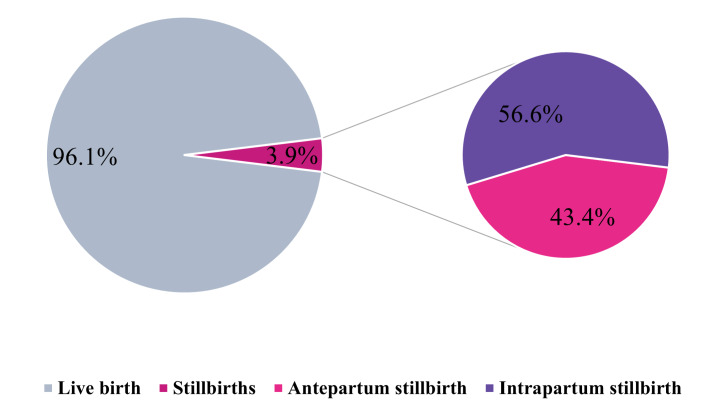
Overall pregnancy outcomes among eight district hospitals of Bangladesh.

Among 15 529 mothers, 96.1% had live births and 3.9% experienced stillbirths. Among all stillbirths, 43.4% were antepartum, and 56.6% were intrapartum (**Figure 1**). At the facility level, stillbirth prevalence varied widely across facilities, ranging from 0.9% in Bagerhat DH to 6.6% in Sunamganj DH, indicating substantial inter-facility heterogeneity. The highest stillbirth percentages were observed in Sunamganj DH (6.6%), followed by Bhola DH (5.8%) and Gaibandha DH (5.0%), while the lowest was in Bagerhat DH (0.9%). In most facilities, intrapartum stillbirths constituted the majority, particularly in Khagrachari DH (70.7%), Sunamganj DH (69.8%), and Bogura DH (69.2%). In contrast, antepartum stillbirths predominated in Munshiganj DH (72.2%) and Netrokona DH (60.7%) (Figure S4 in the **Online Supplementary Document**).

Stillbirths were significantly associated with maternal age, with the highest intrapartum stillbirth percentage found among mothers aged > 34 years (3.4%) (*P* < 0.05). Preterm births showed a markedly higher burden of stillbirth, with 10.2% antepartum and 6.8% intrapartum stillbirths, compared with term and post-term births (*P* < 0.05). Lack of receiving ANC was associated with a substantially higher percentage of intrapartum stillbirths (5.9%) compared with mothers who received at least one ANC visit (1.9%, *P* < 0.05). Non-use of the partograph was associated with nearly a 2-fold higher stillbirth burden, particularly for intrapartum stillbirths (3.6 *vs.* 1.6%, *P* < 0.05). Low birthweight (<2.5 kg) had the highest stillbirth burden, with 4.1% antepartum and 5.5% intrapartum stillbirths, significantly higher than normal-weight births (*P* < 0.05) (**Table 1**).

**Table 1:** Distribution of maternal and neonatal characteristics by pregnancy outcome

Characteristics	Live births, N (%)	Antepartum stillbirths, N (%)	Intrapartum stillbirths, N (%)	*P*-value
**Age, in years**				
< 20	1921 (96.7)	20 (1.0)	45 (2.3)	<0.05
20–24	5912 (96.3)	98 (1.6)	129 (2.1)	
25–29	3983 (96.1)	75 (1.8)	87 (2.1)	
30–34	2090 (95.9)	46 (2.1)	43 (2.0)	
> 34	1016 (94.5)	22 (2.1)	37 (3.4)	
Missing	5 (100.0)	0 (0.0)	0 (0.0)	
**Gestational age**				
Preterm	1288 (83.0)	159 (10.2)	106 (6.8)	<0.05
Term	11 156 (97.5)	80 (0.7)	202 (1.8)	
Post-term	2483 (97.8)	22 (0.9)	33 (1.3)	
**Received ANC**				
Yes (≥ 1 visit)	13 706 (96.5)	233 (1.6)	263 (1.9)	<0.05
No	1221 (92.0)	28 (2.1)	78 (5.9)	
**Partograph used**				
Yes	10 783 (97.4)	115 (1.0)	179 (1.6)	<0.05
No	4144 (93.1)	146 (3.3)	162 (3.6)	
**Sex of newborn**				
Male	7727 (97.0)	106 (1.3)	137 (1.7)	<0.05
Female	7149 (96.8)	83 (1.1)	150 (2.0)	
Missing	51 (28.8)	72 (40.7)	54 (30.5)	
**Birthweight**				
< 2.5	1209 (90.4)	55 (4.1)	73 (5.5)	<0.05
2.5–3.5	11 899 (98.3)	65 (0.5)	138 (1.2)	
3.6–4.5	1684 (98.6)	9 (0.5)	15 (0.9)	
> 4.5	66 (95.7)	2 (2.9)	1 (1.5)	
Missing	69 (22.0)	130 (41.5)	114 (36.4)	
**Total**	14 927 (96.1)	261 (1.7)	341 (2.2)	

ANC – antenatal care

**Figure 2:**
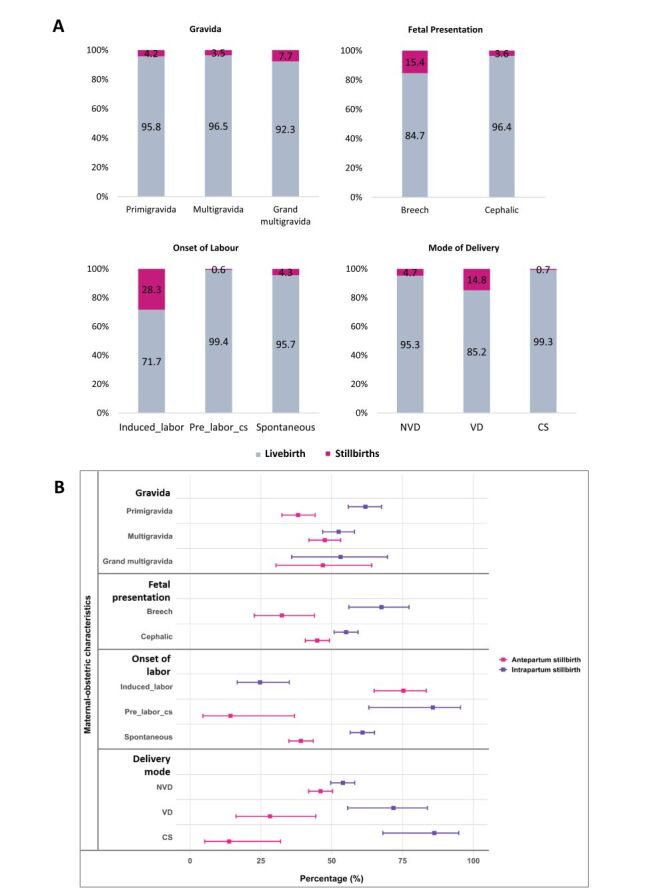
**Panel A**. Percentage of stillbirths among total births by maternal and obstetric characteristics. **Panel B**. Proportion percentage of antepartum and intrapartum stillbirths with 95% CI. CI – confidence interval.

Stillbirths were highest among grand multigravida mothers (7.7%), breech presentations (15.4%), induced labour (28.3%), and VD (14.8%) (**Figure 2**, Panel A). The distribution of antepartum and intrapartum stillbirths did not differ significantly by gravidity (*P* = 0.071). Across all gravida groups, intrapartum stillbirths predominated among primigravida mothers, accounting for 61.9% (95% confidence interval (CI) = 55.9–67.6), while antepartum stillbirths accounted for 38.1% (95% CI = 32.4–44.1). Foetal presentation was significantly associated with the timing of stillbirth, with breech presentation showing a higher percentage of intrapartum stillbirths (67.6%, 95% CI = 56.0–77.3) compared with cephalic presentation (55.1%, 95% CI = 50.8–59.3). Onset of labour was significantly associated with the timing of stillbirth (*P* < 0.05). Induced labour was predominantly associated with antepartum stillbirths (75.3%, 95% CI = 65.0–83.4), whereas pre-labour CS (85.7%, 95% CI = 63.1–95.5) and spontaneous labour (60.9%, 95% CI = 56.5–65.1) were dominated by intrapartum stillbirths. Mode of delivery was significantly associated with stillbirth timing, with the highest percentage of intrapartum stillbirths observed among CSs (86.2%, 95% CI = 68.1–94.8) and VDs (71.8%, 95% CI = 55.6–83.8) (**Figure 2**, Panel B).

**Figure 3:**
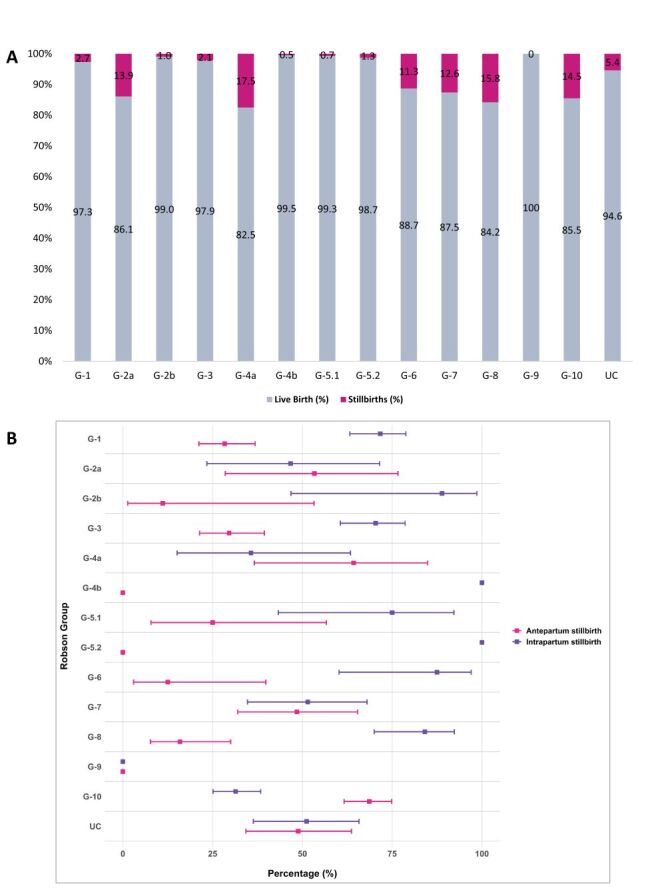
**Panel A.** Percentage of stillbirths among total births by Robson Classification System. **Panel B**. Proportion percentage of antepartum and intrapartum stillbirths with 95% CI. CI – confidence interval.

The stillbirth percentage varied widely across Robson groups, ranging from 0.0% in Group 9 to 17.5% in Group 4a. The highest stillbirth burdens were observed in Group 4a (17.5%), followed by Group 8 (15.8%), Group 10 (14.5%), and Group 2a (13.9%), whereas very low percentages were seen in Group 4b (0.5%) and Group 5.1 (0.7%) (**Figure 3**, Panel A). The distribution of antepartum and intrapartum stillbirthsdiffered significantly across Robson groups (*P* < 0.05). Intrapartum stillbirths predominated in most Robson groups, including G-1 (71.7%, 95% CI = 63.2–78.8), G-3 (70.4%, 95% CI = 60.6–78.6), G-6 (87.5%, 95% CI = 60.2–97.0), and G-8 (84.1%, 95% CI = 70.0–92.3). Groups G-4b and G-5.2 showed exclusively intrapartum stillbirths (100%), though with small numbers. In contrast, Group G-10 was dominated by antepartum stillbirths (68.6%, 95% CI = 61.6–74.9), with a lower percentage of intrapartum stillbirths (31.4%, 95% CI = 25.1–38.4) (**Figure 3**, Panel B).

Substantial inter-facility variation was observed in the timing of stillbirths across Robson groups. In Group 1, intrapartum stillbirths predominated in most facilities, ranging from 66.7% in Bagerhat to 100.0% in Gaibandha and Munshiganj, while antepartum stillbirths reached 48.2% in Netrokona. Group 10 showed a predominantly antepartum pattern across most hospitals, including Bagerhat (100%), Bhola (74.1%), Bogura (62.5%), Gaibandha (72.4%), Munshiganj (100%), and Netrokona (77.8%). Groups G-6, G-8, and G-3 were largely intrapartum-dominated across facilities, with 100% intrapartum stillbirths reported for Group 6 in Bogura, Gaibandha, and Khagrachari, and for Group 8 in Bagerhat, Bogura, Gaibandha, and Khagrachari (Table S4 in the **Online Supplementary Document**).

## DISCUSSION

This study revealed a significant challenge to the quality of perinatal care in secondary-level DHs in Bangladesh. The overall stillbirth rate was 3.9%, with intrapartum stillbirths constituting the majority at 56.6%, indicating a substantial proportion of preventable deaths related to labour and delivery management. These findings suggest that stillbirths in district hospitals are concentrated in clinically high-risk obstetric contexts, reflecting both maternal-foetal vulnerability and gaps in health-system performance.

The observed stillbirth rate (n/N = 39/1000) is substantially higher than the national estimate of n/N = 21/1000 total births reported in the Bangladesh Demographic Health Survey 2022 [24]. This difference is expected, as district hospitals serve as referral points for complicated and high-risk pregnancies, whereas national estimates reflect the general population. Our findings are comparable to hospital-based studies in Nigeria (n/N = 38/1000 total births) [25]. These variations likely reflect differences in facility readiness, quality of intrapartum care, referral timeliness, and socio-economic contexts. The consistently high intrapartum contribution across settings suggests that a substantial proportion of stillbirths could be averted through improved labour monitoring, timely recognition of complications, and prompt emergency obstetric response by skilled attendants [20,26,27].

The higher burden of stillbirth among older mothers reflects the well-established obstetric risks associated with advanced maternal age, including increased rates of congenital anomalies, hypertensive disorders, diabetes, and placental dysfunction, all of which elevate the risk of foetal death [20,28–33]. The strong link between preterm birth and stillbirth is biologically plausible, as preterm foetuses are especially vulnerable to both chronic maternal conditions and acute intrapartum insults [34]. The occurrence of stillbirths among infants with normal birthweight further emphasises that a substantial proportion of these deaths are potentially preventable with timely recognition of complications and prompt intrapartum intervention [1]. Intrapartum stillbirths are commonly driven by events such as obstructed or prolonged labour, umbilical cord complications, placental abruption, and delays in responding to foetal distress, which result in intrapartum hypoxia and death [35]. Although ANC coverage is generally high in Bangladesh, gaps in the quality and effectiveness of ANC limit its protective impact on stillbirth prevention [36]. Inadequate labour monitoring, particularly poor or inconsistent use of the partograph, further compromises timely detection of labour abnormalities and remains a critical barrier to preventing intrapartum stillbirths [37].

Breech presentation is a recognised risk factor for stillbirth and is consistently associated with poorer pregnancy outcomes compared with cephalic presentation, as seen in other studies [38]. In district hospitals, the continued occurrence of stillbirth among breech deliveries highlights challenges in timely decision-making, availability of CS services, and adherence to standard clinical guidelines. Most stillbirths in low-resource settings are delivered vaginally, as reported in earlier studies [39,40]. Although caesarean delivery appeared less associated with stillbirth in our setting, evidence from Ethiopia has shown lower odds of stillbirth with VD, likely reflecting differences in case selection and timing of intervention [41]. Regular, good-quality ANC is essential for reducing the risk of stillbirth through early detection of warning signs, timely referral, and appropriate planning of the mode of delivery [36,42]. Patterns of labour management also influence stillbirth outcomes. Induction of labour is commonly performed after confirmed foetal death, as spontaneous labour usually occurs within one to two weeks [43–45]. While induction is a life-saving intervention for high-risk pregnancies, unnecessary early-term induction can be harmful for otherwise healthy infants [46,47]. After 28 weeks of gestation, the optimal mode of delivery for stillbirth remains uncertain; however, caesarean delivery should generally be avoided in the absence of maternal indications, in line with American College of Obstetricians and Gynecologists (ACOG) recommendations for standard obstetric practice [44,47,48].

Use of the Robson classification system provided important group-specific insights into the timing and clinical context of stillbirths. The concentration of antepartum deaths in preterm singleton pregnancies reflects the strong link between preterm birth and maternal complications such as hypertension and placental insufficiency, which are well-known contributors to foetal death before labour [18,19,49]. In contrast, the predominance of intrapartum stillbirths in breech, multiple, and spontaneous labour groups points to gaps in labour monitoring, foetal surveillance, and timely clinical decision-making for both nulliparous and multiparous women. High intrapartum risk in breech and multiple pregnancies reinforces the need for close monitoring and timely surgical intervention in these high-risk groups [18]. The substantial intrapartum contribution from otherwise lower-risk spontaneous labour onset groups further highlights persistent deficiencies in partograph use, early detection of foetal distress, and prompt obstetric response in high-volume settings, leading to potentially preventable deaths [48,50,51]. The higher antepartum stillbirth burden in some facilities likely reflects delayed referral, limited capacity to manage preterm and high-risk pregnancies, inconsistent ANC, and inadequate monitoring of maternal complications [9,18,52,53]. Intrapartum stillbirths, on the other hand, point to gaps in labour monitoring, delayed emergency response, and challenges in managing complicated labour and foetal presentations [50,53–55]. These findings emphasise the need to strengthen referral systems, ensure consistent intrapartum monitoring, and reinforce adherence to labour management protocols, supported by continuous training and supervision of health care providers.

These findings demonstrate that the Robson system is useful beyond CS audits, allowing facilities to identify both high-risk and high-volume groups for targeted quality-improvement actions. Ongoing staff training, strengthened triage, and routine audit-and-feedback using Robson groups are essential to improve accountability and quality of care. Facility-level variation in these patterns further indicates that localised, context-specific responses are required rather than a single uniform strategy. Reducing stillbirths in Bangladesh requires strengthening both ANC (for early detection of complications such as hypertensive disorders, infections, and growth restriction) and intrapartum care (for timely monitoring and intervention in obstructed labour, malpresentation, and foetal distress). Expanding skilled birth attendance, enforcing consistent use of partographs, and ensuring timely access to emergency obstetric care, including caesarean delivery when indicated, are priorities. Targeted improvements should focus on Robson's high-risk groups, particularly Groups 5–10. Investments in health worker training, referral systems, and community awareness are also needed to ensure equitable access, especially in rural and underserved areas.

### Strengths and limitations

This study has several strengths. It is the first to apply the Robson classification system to stillbirths in Bangladesh, linking it to maternal-obstetric characteristics across multiple district hospitals, providing geographical diversity. The use of prospectively collected facility data reduces recall bias and improves accuracy. Group-specific analysis enabled identification of obstetric subgroups most vulnerable to stillbirth, enhancing the programmatic relevance of the findings. Several limitations should also be acknowledged. Data were restricted to secondary-level public hospitals and may not represent private or tertiary settings, where care-mix and case patterns may differ. Antepartum risk factors before admission and community-level stillbirths were not captured. The analysis was descriptive and did not explore causal pathways such as health system delays or provider-level practices. The study data were limited to eight district hospitals, which capture only a subset of births – primarily complicated or referred cases. As a result, the stillbirth rates presented here reflect a higher-risk population and should not be interpreted as population-level prevalence.

Future research should include tertiary and private facilities to assess generalisability and determine whether similar Robson group patterns exist across different levels of care. Qualitative and mixed-methods studies are needed to understand better referral delays, clinical decision-making, and facility readiness. Operational research evaluating targeted interventions for high-risk Robson groups, such as provider training, improved monitoring technologies, and referral system strengthening, will be critical for accelerating progress toward Sustainable Development Goal 3.2 and ENAP stillbirth targets.

## CONCLUSION

This study demonstrates a substantial stillbirth burden in Bangladeshi district hospitals, with intrapartum deaths exceeding antepartum deaths and clustering within specific high-risk Robson groups. The findings underscore persistent gaps in ANC quality, labour monitoring, and timely obstetric intervention, while also illustrating the value of the Robson Classification System as a practical surveillance and quality-improvement tool for stillbirths. Strengthening the quality of ANC, ensuring consistent use of the partograph, and improving intrapartum monitoring and emergency obstetric response, particularly for high-risk Robson groups, are essential to reducing preventable stillbirths and advancing Bangladesh’s progress toward ENAP and Sustainable Development Goal targets.

## Additional material


Online Supplementary Document
