# Supplementary material for: Stillbirths by maternal-obstetric characteristics and Robson classification system: a cross-sectional study from eight district hospitals in Bangladesh
Source: J Glob Health. 2026 Mar 13;16:04077. doi: 10.7189/jogh.16.04077 (PMC12983048; doi:10.7189/jogh.16.04077)
Supplement: Online Supplementary Document [file jogh-16-04077-s001.pdf]

Supplement to: Hossain L, Mallick T, Sayeed A, Kader ML, Al-Zubayer MA, Azrin F, Mahmood HR, Jahan MS, Dewan F, Shovon MMHK, Saha N, Rahman F, Sajib MRUZ, Hawlader MDH, Mitra D, Sarker MMH, Arifeen SE, Rahman AE, Ahmed A. Stillbirths by maternal-obstetric characteristics and Robson classification system: a cross-sectional study from eight district hospitals in Bangladesh. J Glob Health 2026;16:04077.

Text S1. Explanation of authorship change statement

Following the initial submission of this manuscript, author Md. Lutful Kader was added to the author list. This change was made in recognition of his significant contributions during the revision process. Specifically, he contributed to data analysis and interpretation, participated in the critical revision of the manuscript, and provided substantial input in addressing the reviewers' comments. All authors have agreed to this change in authorship and confirm that author Md. Lutful Kader fulfills the criteria for authorship.

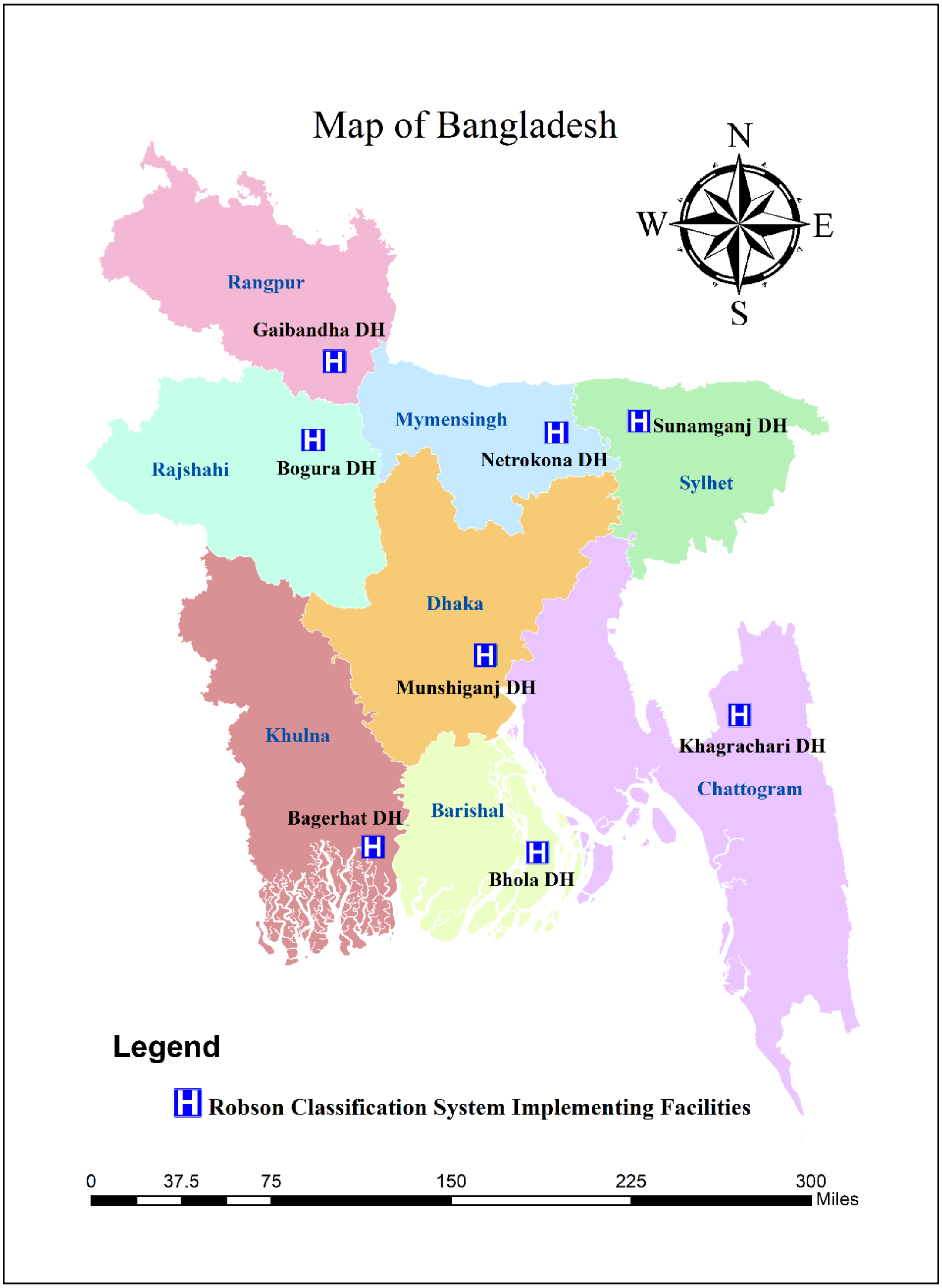

Figure S1: Geographic locations of study facilities across selected district hospitals in Bangladesh

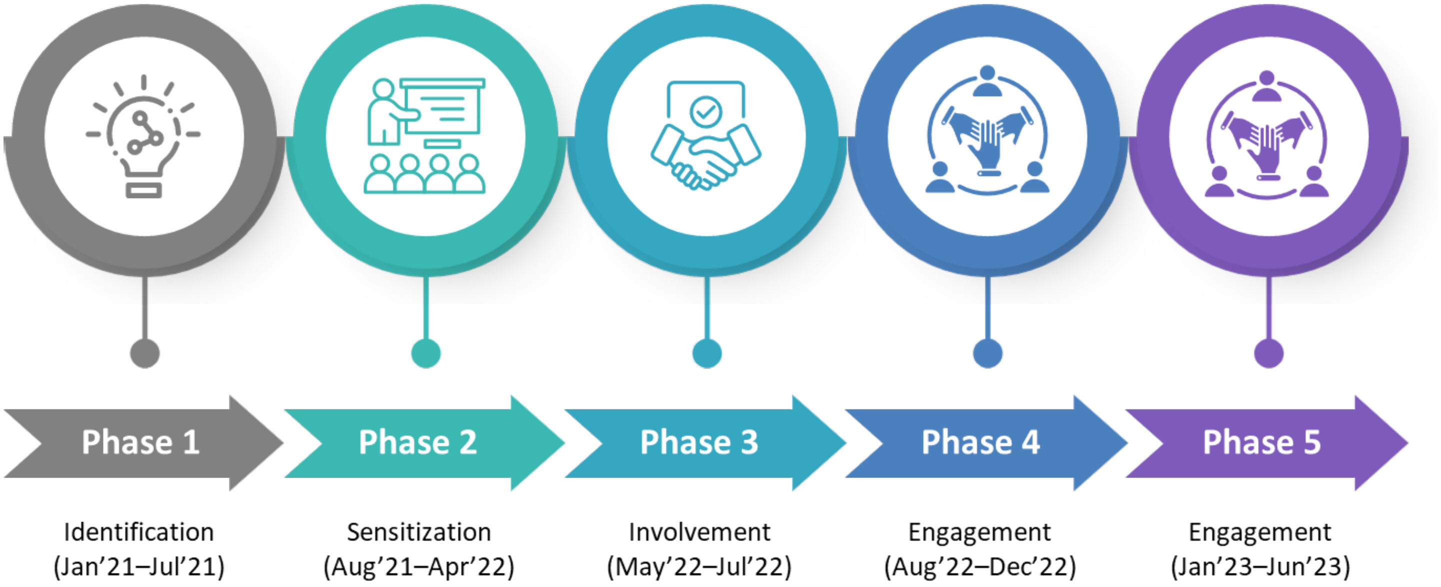

Figure S2: Phases of Implementation of Robson Classification System in selected 8 DHs

Table S1: Robson Classification System with Subgroups and Unclassified Category

Robson Group	Description
**Group 1 (G-1)**	Nulliparous, singleton, cephalic term baby, spontaneous labour
**G-2a**	Nulliparous, singleton, cephalic term baby, labour induced
**G-2b**	Nulliparous, singleton, cephalic term baby, pre-labour CS
**G- 3**	Multiparous, singleton, cephalic term baby, without a previous CS and in spontaneous labour
**G-4a**	Multiparous singleton, cephalic term baby, without a previous CS and who had labour induced
**G-4b**	Multiparous, singleton, cephalic term baby, without a previous CS and who were delivered by CS before labour
**G-5.1**	Multiparous with history of previous one CS, singleton, cephalic term baby
**G-5.2**	Multiparous with history of previous ≥2 CS, singleton, cephalic term baby
**G-6**	Nulliparous, singleton with breech presentation
**G-7**	Multiparous, singleton with breech presentation
**G-8**	Multiple pregnancies including previous CS
**G-9**	All singleton, transverse or oblique lie including previous CS
**G-10**	All singleton cephalic, preterm including previous CS
**Unclassified (UC)**	Cases with missing or inconsistent data that do not fit into any group

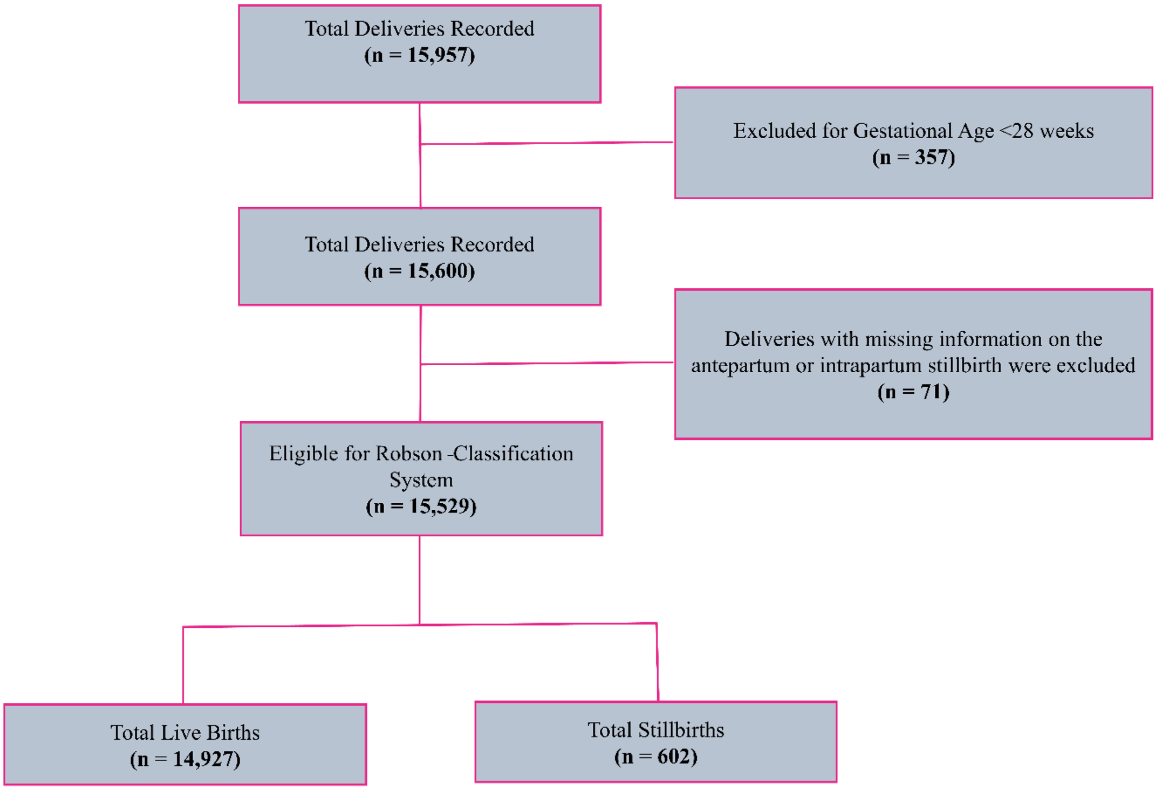

Figure S3: Flowchart Showing Inclusion of Study Participants

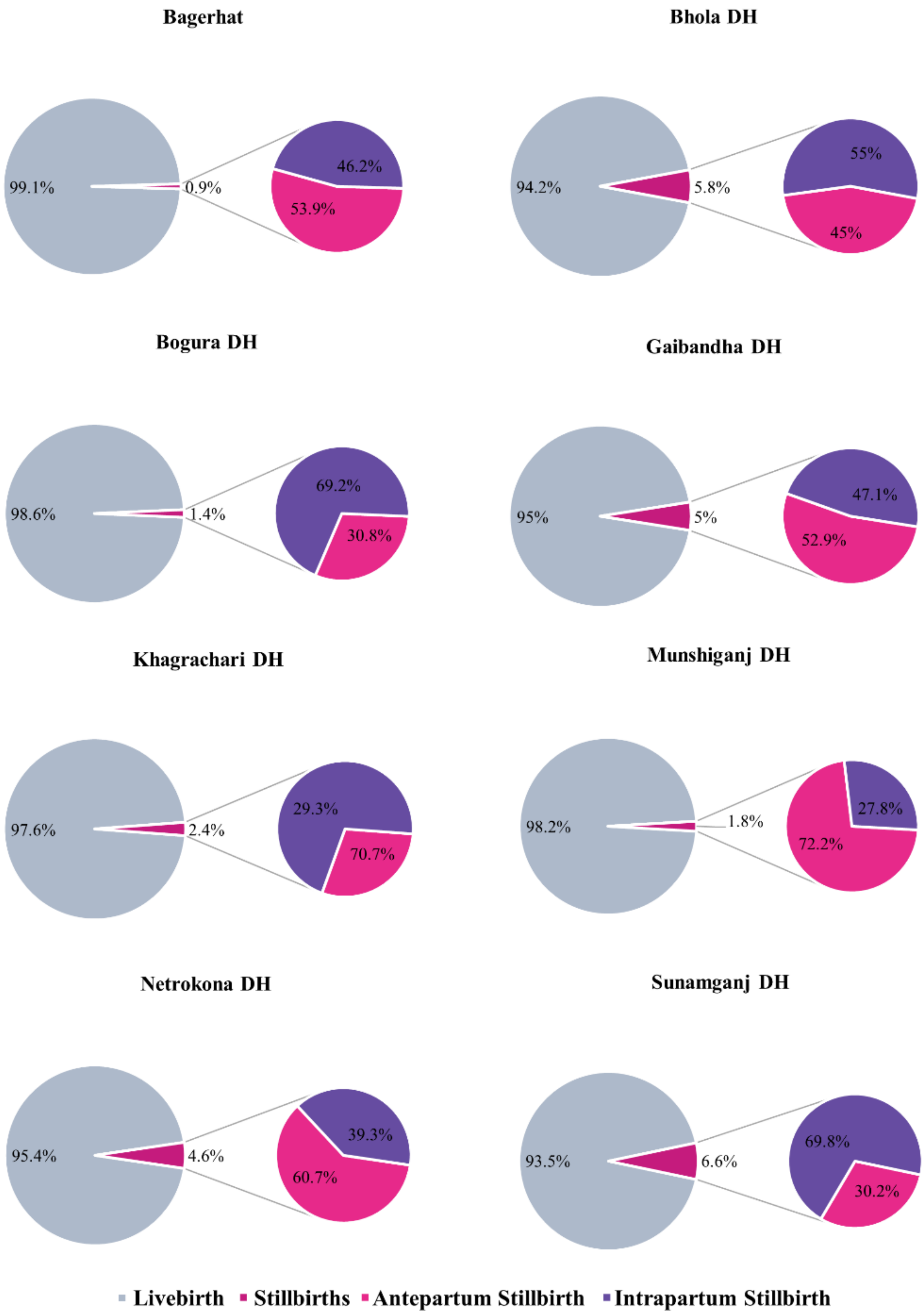

Figure S4: Facility-Specific Pregnancy Outcomes in Eight District Hospitals of Bangladesh

Table S2: Association of Maternal–Obstetric Characteristics with Stillbirths

Variables	Antepartum Stillbirth n (%)	Intrapartum Stillbirth n (%)	P value
**Gravida**			
Primigravida	101 (38.1)	164 (61.9)	0.071
Multigravida	145 (47.5)	160 (52.5)	
Grand multigravida	15 (46.9)	17 (53.1)	
**Fetal presentation**			
Breech	24 (32.4)	50 (67.6)	$p < 0.05$*
Cephalic	237 (44.9)	291 (55.1)	
**Onset of Labor**			
Induced labor	64 (75.3)	21 (24.7)	$p < 0.05$*
Pre-labor CS	3 (14.3)	18 (85.7)	
Spontaneous	194 (39.1)	302 (60.9)	
**Delivery mode**			
NVD	246 (46.1)	288 (53.9)	$p < 0.05$*
VD	11 (28.2)	28 (71.8)	
CS	4 (13.8)	25 (86.2)	

Table S3: Association of Robson Classification System with Stillbirth Types

Robson Groups	Antepartum stillbirth n (%)	Intrapartum Stillbirths	P value
**G-1**	36 (28.4)	91 (71.7)	$p < 0.05$
**G-2a**	8 (53.3)	7 (46.7)	
**G-2b**	1 (11.1)	8 (88.9)	
**G-3**	29 (29.6)	69 (70.4)	
**G-4a**	9 (64.3)	5 (35.7)	
**G-4b**	0 (0.0)	2 (100.0)	
**G-5.1**	3 (25.0)	9 (75.0)	
**G-5.2**	0 (0.0)	1 (100.0)	
**G-6**	2 (12.5)	14 (87.5)	
**G-7**	16 (48.5)	17 (51.5)	
**G-8**	7 (15.9)	37 (84.1)	
**G-9**	0 (0.0)	0 (—)	
**G-10**	129 (68.6)	59 (31.4)	
**UC**	21 (48.8)	22 (51.2)	

Table S4: Distribution of Antepartum stillbirth and Intrapartum stillbirth by Robson groups across eight district hospitals

Robson Group	Bagerhat DH		Bhola DH		Bogura DH		Gaibandha DH		Khagrachari DH		Munshiganj DH		Netrokona DH		Sunamganj DH	
	Antepartum Stillbirth n (%)	Intrapartum Stillbirth n (%)	Antepartum Stillbirth n (%)	Intrapartum Stillbirth n (%)	Antepartum Stillbirth n (%)	Intrapartum Stillbirth n (%)	Antepartum Stillbirth n (%)	Intrapartum Stillbirth n (%)	Antepartum Stillbirth n (%)	Intrapartum Stillbirth n (%)	Antepartum Stillbirth n (%)	Intrapartum Stillbirth n (%)	Antepartum Stillbirth n (%)	Intrapartum Stillbirth n (%)	Antepartum Stillbirth n (%)	Intrapartum Stillbirth n (%)
**G-1**	1 (33.3)	2 (66.7)	7 (26.9)	19 (73.1)	—	—	0 (0.0)	2 (100.0)	5 (50.0)	5 (50.0)	0 (0.0)	1 (100.0)	13 (48.2)	14 (51.9)	10 (17.9)	46 (82.1)
**G-2a**	—	—	—	—	—	—	—	—	1 (50.0)	1 (50.0)	1 (100.0)	0 (0.0)	1 (100.0)	0 (0.0)	2 (40.0)	3 (60.0)
**G-2b**	—	—	0 (0.0)	1 (100.0)	0 (0.0)	1 (100.0)	—	—	—	—	—	—	0 (0.0)	1 (100.0)	1 (100.0)	0 (0.0)
**G-3**	1 (25.0)	3 (75.0)	8 (34.8)	15 (65.2)	0 (0.0)	3 (100.0)	1 (16.7)	5 (83.3)	3 (25.0)	9 (75.0)	1 (100.0)	0 (0.0)	8 (47.1)	9 (52.9)	7 (21.9)	25 (78.1)
**G-4a**	—	—	—	—	1 (100.0)	0 (0.0)	—	—	1 (100.0)	0 (0.0)	1 (50.0)	1 (50.0)	0 (0.0)	1 (100.0)	3 (60.0)	2 (40.0)
**G-4b**	—	—	—	—	—	—	—	—	0 (0.0)	2 (100.0)	—	—	—	—	0 (0.0)	1 (100.0)
**G-5.1**	—	—	—	—	0 (0.0)	3 (100.0)	—	—	0 (0.0)	1 (100.0)	0 (0.0)	2 (100.0)	1 (100.0)	0 (0.0)	2 (40.0)	3 (60.0)
**G-5.2**	—	—	—	—	—	—	—	—	—	—	—	—	—	—	0 (0.0)	1 (100.0)
**G-6**	—	—	1 (33.3)	2 (66.7)	0 (0.0)	1 (100.0)	0 (0.0)	2 (100.0)	0 (0.0)	1 (100.0)	—	—	0 (0.0)	2 (100.0)	1 (14.3)	6 (85.7)
**G-7**	—	—	4 (80.0)	1 (20.0)	2 (50.0)	2 (50.0)	2 (66.7)	1 (33.3)	0 (0.0)	5 (100.0)	—	—	6 (54.6)	5 (45.5)	1 (25.0)	3 (75.0)
**G-8**	0 (0.0)	1 (100.0)	1 (10.0)	9 (90.0)	0 (0.0)	1 (100.0)	0 (0.0)	3 (100.0)	0 (0.0)	3 (100.0)	1 (100.0)	0 (0.0)	1 (20.0)	4 (80.0)	5 (23.8)	16 (76.2)
**G-9**	—	—	—	—	—	—	—	—	—	—	—	—	25 (55.6)	20 (44.4)	—	—
**G-10**	4 (100.0)	0 (0.0)	20 (74.1)	7 (25.9)	5 (62.5)	3 (37.5)	21 (72.4)	8 (27.6)	4 (30.8)	9 (69.2)	8 (100.0)	0 (0.0)	42 (77.8)	12 (22.2)	2 (55.6)	3 (44.4)
**UC (99)**	1 (100.0)	0 (0.0)	7 (53.9)	6 (46.2)	1 (100.0)	0 (0.0)	1 (33.3)	2 (66.7)	1 (20.0)	4 (80.0)	1 (50.0)	1 (50.0)	7 (63.6)	4 (36.4)	2 (28.6)	5 (71.4)
